# ICG-Fluorescence Imaging for Margin Assessment During Minimally Invasive Colorectal Liver Metastasis Resection

**DOI:** 10.1001/jamanetworkopen.2024.6548

**Published:** 2024-04-19

**Authors:** Friso B. Achterberg, Okker D. Bijlstra, Maxime D. Slooter, Babs G. Sibinga Mulder, Mark C. Boonstra, Stefan A. Bouwense, Koop Bosscha, Mariëlle M. E. Coolsen, Wouter J. M. Derksen, Michael F. Gerhards, Paul D. Gobardhan, Jeroen Hagendoorn, Daan Lips, Hendrik A. Marsman, Babs M. Zonderhuis, Lissa Wullaert, Hein Putter, Jacobus Burggraaf, J. Sven D. Mieog, Alexander L. Vahrmeijer, Rutger-Jan Swijnenburg

**Affiliations:** 1 Department of Surgery, Leiden University Medical Center, Leiden, the Netherlands; 2 Department of Surgery, Amsterdam University Medical Center, University of Amsterdam, Amsterdam, the Netherlands; 3 Department of Surgery, Amsterdam University Medical Center, Vrije Universiteit Amsterdam, Amsterdam, the Netherlands; 4 Cancer Center Amsterdam, Amsterdam, the Netherlands; 5 NUTRIM School of Nutrition and Translational Research in Metabolism, Maastricht University Medical Center, Maastricht, the Netherlands; 6 Department of Surgery, Jeroen Bosch Ziekenhuis, Den Bosch, the Netherlands; 7 Department of Surgery, Maastricht University Medical Center, Maastricht, the Netherlands; 8 Department of Surgery, St. Antonius Hospital, Nieuwegein/Regionaal Academisch Kankercentrum Utrecht, Utrecht, the Netherlands; 9 Department of Surgery, Onze Lieve Vrouwe Gasthuis, Amsterdam, the Netherlands; 10 Department of Surgery, Amphia Ziekenhuis, Breda, the Netherlands; 11 Department of Surgery, University Medical Center Utrecht/Regionaal Academisch Kankercentrum Utrecht, Utrecht, the Netherlands; 12 Department of Surgery, Medisch Spectrum Twente, Enschede, the Netherlands; 13 Department of Medical Statistics and Bioinformatics, Leiden University Medical Center, Leiden, the Netherlands; 14 Centre for Human Drug Research, Leiden, the Netherlands; 15 Department of Surgical Oncology, Erasmus Medical Center, Rotterdam, the Netherlands

## Abstract

**Importance:**

Unintended tumor-positive resection margins occur frequently during minimally invasive surgery for colorectal liver metastases and potentially negatively influence oncologic outcomes.

**Objective:**

To assess whether indocyanine green (ICG)–fluorescence–guided surgery is associated with achieving a higher radical resection rate in minimally invasive colorectal liver metastasis surgery and to assess the accuracy of ICG fluorescence for predicting the resection margin status.

**Design, Setting, and Participants:**

The MIMIC (Minimally Invasive, Indocyanine-Guided Metastasectomy in Patients With Colorectal Liver Metastases) trial was designed as a prospective single-arm multicenter cohort study in 8 Dutch liver surgery centers. Patients were scheduled to undergo minimally invasive (laparoscopic or robot-assisted) resections of colorectal liver metastases between September 1, 2018, and June 30, 2021.

**Exposures:**

All patients received a single intravenous bolus of 10 mg of ICG 24 hours prior to surgery. During surgery, ICG-fluorescence imaging was used as an adjunct to ultrasonography and regular laparoscopy to guide and assess the resection margin in real time. The ICG-fluorescence imaging was performed during and after liver parenchymal transection to enable real-time assessment of the tumor margin. Absence of ICG fluorescence was favorable both during transection and in the tumor bed directly after resection.

**Main Outcomes and Measures:**

The primary outcome measure was the radical (R0) resection rate, defined by the percentage of colorectal liver metastases resected with at least a 1 mm distance between the tumor and resection plane. Secondary outcomes were the accuracy of ICG fluorescence in detecting margin-positive (R1; <1 mm margin) resections and the change in surgical management.

**Results:**

In total, 225 patients were enrolled, of whom 201 (116 [57.7%] male; median age, 65 [IQR, 57-72] years) with 316 histologically proven colorectal liver metastases were included in the final analysis. The overall R0 resection rate was 92.4%. Re-resection of ICG-fluorescent tissue in the resection cavity was associated with a 5.0% increase in the R0 percentage (from 87.4% to 92.4%; *P* < .001). The sensitivity and specificity for real-time resection margin assessment were 60% and 90%, respectively (area under the receiver operating characteristic curve, 0.751; 95% CI, 0.668-0.833), with a positive predictive value of 54% and a negative predictive value of 92%. After training and proctoring of the first procedures, participating centers that were new to the technique had a comparable false-positive rate for predicting R1 resections during the first 10 procedures (odds ratio, 1.36; 95% CI, 0.44-4.24). The ICG-fluorescence imaging was associated with changes in intraoperative surgical management in 56 (27.9%) of the patients.

**Conclusions and Relevance:**

In this multicenter prospective cohort study, ICG-fluorescence imaging was associated with an increased rate of tumor margin–negative resection and changes in surgical management in more than one-quarter of the patients. The absence of ICG fluorescence during liver parenchymal transection predicted an R0 resection with 92% accuracy. These results suggest that use of ICG fluorescence may provide real-time feedback of the tumor margin and a higher rate of complete oncologic resection.

## Key Points

**Question:**

Is near-infrared fluorescence imaging with indocyanine green (ICG) associated with improved oncologic resections in patients undergoing minimally invasive resections of colorectal liver metastases?

**Findings:**

In this cohort study of 201 adults with 316 histologically proven colorectal liver metastases, the overall rate of complete tumor resection was 92.4%. The absence of ICG fluorescence during parenchymal transection predicted a complete tumor resection (R0) with 92% accuracy.

**Meaning:**

The findings of this study suggest that ICG fluorescence may provide surgeons with real-time feedback of the tumor margin during minimally invasive surgery for colorectal liver metastases and may increase the percentage of complete oncologic resections.

## Teaser

This cohort study of patients undergoing minimally invasive colorectal liver metastasis resections assesses the use of indocyanine green–fluorescence imaging to achieve a higher radical resection rate and greater accuracy in estimating the resection tumor-margin status.

## Introduction

Approximately 50% of patients with colorectal cancer are diagnosed with colorectal liver metastasis (CRLM) during the course of their disease.^1,2^ For CRLM, partial hepatectomy with tumor-negative margins of at least 1 mm is considered a radical (R0) oncologic resection, resulting in improved local disease control and a 5-year survival of over 50%.^1,2^ Although the percentage of R0 resections has improved over the past decades, some studies still report unintended microscopically incomplete (R1) margins (<1 mm) in 14% to 27% of patients.^3,4^ Although unintended (parenchymal) R1 resections lead to higher local recurrence rates compared with R0 resections, intended (vascular) R1 resections, particularly after induction chemotherapy, result in similar local disease control and are widely accepted as resections with curative intent.^5^ One therefore aims to maintain sufficient margin for a complete oncologic resection, while sparing as much liver parenchyma as possible.

With the introduction of laparoscopic liver surgery, and, more recently, robot-assisted liver surgery, the number of minimally invasive procedures for CRLM has rapidly increased.^6,7^ Minimally invasive surgery has shown benefits regarding length of hospital stay and complication rates, with similar oncologic outcomes.^3^ However, minimally invasive procedures preclude the surgeon’s tactile feedback, making tumor identification, resection planning, and tumor-margin assessment challenging. Therefore, intraoperative ultrasonography (IOUS) is frequently used for tumor identification and resection planning. Given the negative effect of a positive resection margin on oncologic outcomes, it can be argued that there is an unmet clinical need to assess the tumor margin more accurately during surgery. In the event of a suspected tumor-positive resection, the surgeon may increase the margin if feasible by adapting the transection plane or resecting additional tissue.

Novel imaging methods like near-infrared (NIR) fluorescence imaging with indocyanine green (ICG) might compensate for the lack of tactile feedback and serve as an adjunct to IOUS.^8,9^ Most clinically available laparoscopic and robotic imaging systems are suitable for or are already equipped with an NIR light engine for fluorescence imaging. The nonionizing NIR light can penetrate tissue up to plus or minus 8 mm.^10^ Moreover, ICG-fluorescence imaging is currently widely applied in hepatopancreatiobiliary surgery for multiple indications (eg, fluorescence cholangiography; liver segmental perfusion; and, more recently, tumor identification).^11^ For CRLM identification, ICG is intravenously administered 1 to 14 days before surgery, resulting in a rim-shaped enhancement pattern surrounding the tumor tissue.^12,13,14,15,16,17^ After intravenous administration, ICG binds to albumin and is rapidly cleared from the blood by the liver and excreted solely by the biliary tract. However, entrapment of the fluorescent contrast agent inside cholestatic hepatocytes surrounding the CRLM results in the rim-shaped fluorescent pattern, leading to the detection of small occult lesions in up to 12% of patients.^12,13,18,19^

The application of ICG-fluorescence imaging for intraoperative tumor-margin assessment has been reported by multiple groups.^13,14,16^ The presence of ICG fluorescence at the parenchymal transection plane (ie, the dorsal side of the resection specimen) indicates a too-close proximity to the tumor tissue and, therefore, a potential R1 resection margin.^20,21^ Conversely, absence of an ICG-fluorescence signal may indicate a safe margin between the resection plane and the tumor, allowing for intraoperative confirmation of an R0 resection. Whereas regular histopathologic assessment of the surgical specimen may take up to 10 days, immediate information on tumor margin allows the surgeon to perform an immediate re-resection of the surgical cavity, thereby potentially improving oncologic outcome.^22^ In this prospective cohort study (the Minimally Invasive, Indocyanine-Guided Metastasectomy in Patients With Colorectal Liver Metastases [MIMIC] trial), we implemented ICG-fluorescence–guided minimally invasive liver surgery in 8 Dutch liver surgery centers and investigated the diagnostic accuracy of real-time resection margin assessment and the association with R0 resection rates for CRLM surgery.

## Methods

### Study Design

The MIMIC trial was designed as a single-arm prospective cohort study. The study protocol was approved by the medical ethics committee Leiden-Den Haag Delft. The study was registered in the Dutch Trial Register (NL7674) and conducted according to the World Medical Association Declaration of Helsinki involving the ethical principles for medical research involving human subjects.^23^ This study followed the Strengthening the Reporting of Observational Studies in Epidemiology (STROBE) reporting guideline. All participating centers were required to use the fluorescence imaging agent indocyanine green (Diagnostic Green, Verdye GmbH) as part of their hospital’s protocol to identify additional CRLM in patients undergoing liver surgery. All patients provided oral informed consent.

### Patients

Patients 18 years and older and scheduled to undergo minimally invasive, either laparoscopic or robot-assisted ICG-fluorescence–guided resections for CRLM were eligible for inclusion. From September 1, 2018, to June 30, 2021, patients were approached for enrollment by their clinician. The decision to perform either laparoscopic or robot-assisted surgery was surgeon dependent and based on the availability of the imaging system per clinic. Patients with an allergy to iodine or who previously had an allergic reaction after ICG administration were excluded. Also, patients with recurrent CRLM at the site of previous ablation or resection were excluded, given the formation of a deviating rim-enhancement pattern in the regenerating tissue at the site of the previous intervention. Patients with newly developed recurrent CRLM without relation to previous ablation or resection sites were eligible for inclusion.

### Treatment Variables

All patients underwent the standard diagnostic workup according to the national standard, including standard liver tests and serum carcinoembryonic antigen levels, 4-phase computed tomography, and/or magnetic resonance imaging with liver-specific contrast. Baseline characteristics of the primary colorectal tumor (eg, location, treatment, TNM stage), diagnostic workup of the current CRLM episode (number of lesions, size, type of scan, and Fong clinical risk score), surgical parameters (planned procedure type, executed procedure type, procedure time, and blood loss), length of hospital stay, postoperative complications, pathology reports, and short-term oncologic follow-up were registered in the electronic data capture system (Castor).

### Intraoperative Fluorescence Imaging

Approximately 24 hours prior to surgery, 10 mg (2 mL) of a 5-mg/mL bolus injection of ICG was administered intravenously, as described previously.^12,17^ Participating centers were allowed to only use 1 of the following fluorescence imaging systems optimized for ICG-mediated minimally invasive imaging: Image1 S Rubina (Karl Storz), Visera Elite II (Olympus), 1588 or 1688 Advanced Imaging Modalities (Stryker), or Firefly (Intuitive Surgical). All imaging systems used in this study were equipped with an ICG-fluorescence overlay function, which allows for ICG-fluorescence imaging to be used in parallel with regular white light imaging of the laparoscope.

As current clinical imaging systems do not allow for intraoperative quantification of the fluorescence signal, identification of additional lesions and assessment of tumor-positive margins with ICG-fluorescence imaging were performed qualitatively at the surgeon’s discretion. To increase data quality, all participating surgeons (B.G.S.M., S.A.B., K.B., M.M.E.C., W.J.M.D., P.D.G., J.H., D.L., and H.A.M.) were trained in the use of ICG fluorescence prior to the first inclusion using the results and an instructional video of a previous proof-of-concept study by some of us.^16^ Since the imaging was performed qualitatively, no comparison among the imaging systems was performed. The pragmatic design to include commercially available imaging systems was not expected to influence data quality, given that any decision-making was performed at the surgeon’s discretion. Second, a study coordinator (F.B.A., O.D.B., M.D.S., M.C.B., or R.-J.S.) was present during the first 1 to 3 procedures (depending on the surgeon’s previous experience with fluorescence imaging) of the study to proctor qualitative image interpretation for lesion identification and assessment of resection margin status.

### Real-Time Intraoperative Tumor Margin Identification

First, the liver was inspected to identify target lesion or lesions and any additional lesions with regular white light laparoscopy and subsequently with the ICG-fluorescence overlay. Thereafter, all lesions, including more centrally located lesions, were visualized by IOUS. For resection planning, regular white light imaging, IOUS, and an ICG-fluorescence overlay were used as complementary imaging modalities.

During parenchymal transection, ICG-fluorescence overlay imaging was used to identify any ICG-fluorescence signal to provide continuous feedback on the tumor margin. After finalizing the resection, the parenchymal side of the resection specimen was inspected intraoperatively for residual ICG fluorescence to assess the tumor margin. Both the absence (suspected R0 resection) and presence (suspected R1 resection) were registered in the electronic case report form (eCRF). When an R1 resection margin was suspected based on ICG fluorescence, an additional margin was acquired from the surgical cavity when deemed technically feasible and safe at the surgeon’s discretion. Any changes in the surgical management based on ICG-fluorescence imaging defined as (1) identification of fluorescence in the transection plane, leading to an attempt to increase the margin during transection; (2) additional resection of the ICG-fluorescence–positive surgical cavity; and (3) identification of occult CRLM, leading to extended or additional liver resection, were registered in the eCRF.

Final histopathologic assessment of the tumor margin was used as the reference standard and was postoperatively correlated with the surgeon’s intraoperative assessment of the tumor margin. A resection with a tumor-negative margin of at least 1 mm between the resection plane and the tumor’s edge was considered as a radical resection (R0), whereas resections with a margin of less than 1 mm were scored as R1.

### End Points

The primary end point of the study was the R0 resection rate. Secondary end points included the diagnostic accuracy of ICG fluorescence to predict the resection margin status (ie, sensitivity, specificity, positive predictive value [PPV], and negative predictive value [NPV]) using histopathologic assessment as the reference standard, the change in surgical management, the surgeon’s learning curve, and perioperative complications.

Any potential adverse event or complication related to ICG injection was documented. Perioperative complications, scored according to the Clavien-Dindo classification,^24^ during admission and 90 days after discharge were also registered in the eCRF. Admission days in the intensive care unit and total length of hospital stay were recorded. Planned readmission within 90 days after surgery (eg, for resection of the primary tumor or a second intervention of a 2-staged procedure) was not seen as a complication of the hepatic surgery. After completion of the procedure, the surgeons were asked to fill out a questionnaire on the usefulness of ICG-fluorescence imaging and the ability to assess the tumor margin in real time (ie, the quality of ICG-fluorescence imaging) (eAppendices 1 and 2 in Supplement 1).

### Statistical Analysis

Based on a previous large randomized clinical trial, the R1 rate in laparoscopic liver surgery for CRLM was 28%.^3^ The present study aimed to reach a maximum R0 rate of 18% with 1-sided α = .05 and a statistical power of 90%. To reach the primary end point, a sample size of 188 patients was calculated. Considering a dropout rate of 10%, we aimed to include 209 patients in this study.

For statistical analysis, SPSS Statistics, version 25 (IBM Inc), was used. Continuous, not normally distributed values were presented as median (IQR), and normally distributed values as mean (SD). The McNemar test was used to compare paired proportions. First, to avoid confounding factors and enable analysis of the entire cohort, baseline characteristics of laparoscopic liver surgery and robot-assisted liver surgery were compared with the Mann-Whitney test for normally distributed continuous data and the χ^2^ test for not normally distributed categorical data.

To assess the diagnostic accuracy of ICG-fluorescence imaging for positive tumor-margin detection, a per-lesion analysis was performed to calculate sensitivity, specificity, PPV, and NPV, and area under the receiver operating characteristic curves were computed. A 1-sided *P* < .05 was considered statistically significant. An event was defined as a true positive when the ICG-fluorescence signal was found on the parenchymal side of the resection specimen and the lesion was a confirmed R1 resection by histopathologic assessment. An event was defined as a true negative if the ICG-fluorescence signal was negative and histopathologic assessment confirmed an R0 resection. When the ICG-fluorescence signal was found on the parenchymal side of the resection specimen in an R0 resection, the event was defined as false positive. When no ICG-fluorescence signal was found in a histologically confirmed R1 resection, the event was defined as a false negative (eTable 1 in Supplement 1).

Since no literature is yet available describing the learning curve of ICG-fluorescence imaging, a regression analysis was performed comparing experienced and inexperienced centers. For this analysis, a cutoff value of 10 procedures was used.

## Results

### Cohort

**Figure 1.**
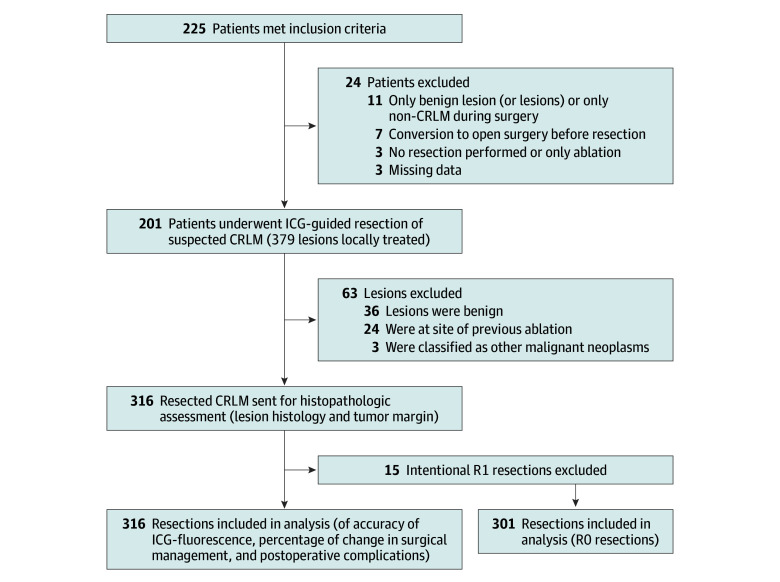
Flow Diagram of Inclusions and Exclusions of Study Patients and Resections CRLM indicates colorectal liver metastasis; ICG, indocyanine green; R0, negative tumor margin; R1, positive tumor margin.

From September 2018 to June 2021, 225 patients were enrolled in the MIMIC trial. Of these, 24 were excluded from the analysis for a variety of reasons, as shown in Figure 1. A total of 201 patients (median age, 65 years [IQR, 57-72 years]; 85 female [42.3%] and 116 male [57.7%]), with 436 liver lesions suspected of being CRLM (mean [SD], 2.17 [2.30]), were included in the final analysis. Of all suspected colorectal liver metastases, 379 were locally treated, 355 by resection and 24 by thermal ablation. The remaining lesions were either part of a 2-staged procedure (and therefore not included in this study) or diagnosed as benign lesions during the procedure and not registered as CRLM. Baseline and patient characteristics are summarized in Table 1. Most patients (130 [64.7%]) underwent robot-assisted surgery, which has seen wide implementation in the Netherlands. Baseline characteristics, intraoperative, and postoperative variables were comparable for patients operated on by robot-assisted surgery and laparoscopic surgery (eTable 2 in Supplement 1). Most primary tumors (n = 2 missing patients) were located in the colon (n = 127 [63.8%]), whereas 72 patients (36.2%) had primary rectal cancer. In total, 58 patients (28.9%) received neoadjuvant chemotherapy for CRLM. After histopathologic analysis, 316 (89.0%) of the 355 resected lesions were confirmed as CRLM, 3 lesions (0.1%) were classified as other malignant neoplasms, and 36 (10.1%) were benign lesions.

**Table 1.** Baseline Patient Characteristics and Perioperative Data

Characteristics	Participants, No. (%) (N = 201)
Age, median (IQR), y	65 (57-72)
Sex	
Female	85 (42.3)
Male	116 (57.7)
Location of primary tumor (n = 199)^a^	
Ascending colon	40 (20.1)
Transverse colon	6 (3.0)
Descending colon	9 (4.5)
Sigmoid	72 (36.2)
Rectum	72 (36.2)
T stage	
T1	8 (4.0)
T2	23 (11.6)
T3	135 (67.8)
T4	33 (16.6)
N stage	
N0	63 (31.8)
N1	90 (45.5)
N2	45 (22.7)
M stage	
M0	99 (49.0)
M1	102 (51.0)
Serum carcinoembryonic antigen, ng/mL, median (IQR)	5.9 (2.7-15.0)
No. of suspected CRLM lesions, mean (SD)	2.17 (2.30)
Size of largest lesion, mm, median (IQR)	22 (14-35)
Neoadjuvant chemotherapy	58 (28.9)
Surgery characteristics, median (IQR)	
Operation time, min	157 (30-450)
Estimated blood loss, mL	150 (50-350)
Hospital stay, d	3 (2-4)
Clavien-Dindo classification^b^	
No complication	169 (84.1)
Low	15 (7.5)
High	11 (5.5)
Missing	6 (3.0)

Abbreviation: CRLM, colorectal liver metastases.

SI conversion: To convert carcinoembryonic antigen to micrograms per liter, multiply by 1.0.

^a^ Data from 2 patients were missing because they were referred from a center outside of the Netherlands, and both patients were unaware of the location of their primary tumor.

^b^ Classifications range from 0 to 5, with 3 to 5 indicting more severe postoperative complications.

### Association of ICG-Fluorescence Imaging With a Tumor-Negative Resection

**Figure 2.**
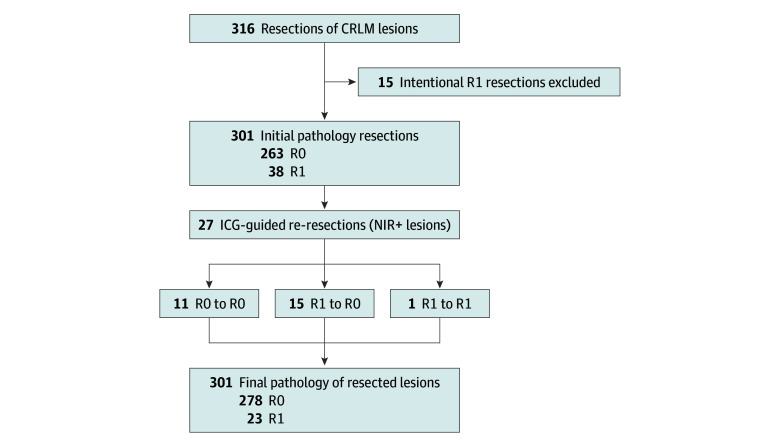
Flow Diagram of Changes in Final Pathology After Re-Resection Per-lesion analysis of all resected and histologically proven colorectal liver metastasis (CRLM) lesions showing an initial radical resection rate of 82.3% and final radical resection rate of 92.4% after additional resection. Based on fluorescence signal with histopathologic assessment as the gold standard, the accuracy of indocyanine green (ICG)–fluorescence imaging as an indicator for resection margins was calculated in the final step. NIR+ indicates positive near-infrared fluorescence signal; R0, negative tumor margin; R1, positive tumor margin.

After exclusion of the 15 vascular R1 resections, 301 resections of CRLM remained for the clinical outcomes analysis. Following the initial resection, resection of additional parenchyma from the surgical cavity was performed in 27 (8.5%) of 316 patients, based on ICG-fluorescence positivity. In 16 (59.3%) of those 27 patients, this re-resection converted the margin status from R1 to R0 on the final pathology, leading to an increase of the R0 rate of 5.0% (from 87.4% to an overall 92.4%; *P* < .001) with a mean (SE) margin of 6.73 (0.497) mm (Figure 2). In 15 of these 16 specimens, no additional malignant tissue was detected and thus increased the margin width. Initial radical resection rates were similar for patients treated with neoadjuvant chemotherapy vs those who did not receive neoadjuvant chemotherapy (86% vs 81%; *P* = .35).

### Diagnostic Accuracy of ICG-Fluorescence Imaging for Assessment

**Figure 3.**
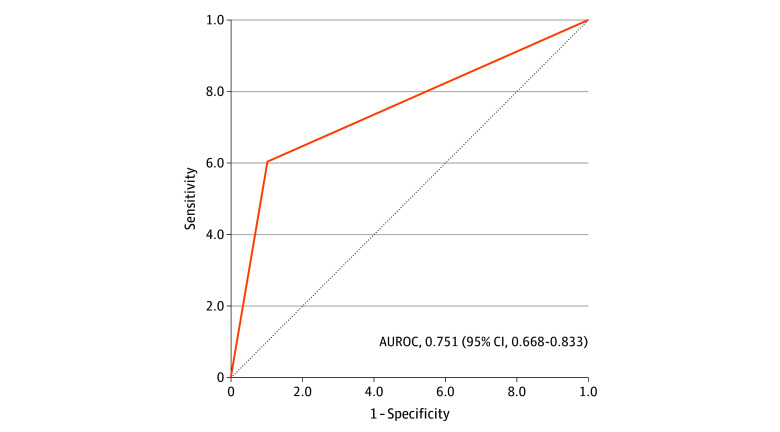
Area Under the Receiver Operating Characteristic Curve (AUROC) for the Accuracy of Indocyanine Green–Fluorescence Imaging

Of the 316 histopathologically confirmed CRLM lesions, 53 (16.8%) of initially resected lesions were classified as R1 (mean [SD] margin, 0.070 [0.210] mm). Of these 53 lesions, 15 (28.3%) were intentional R1 resections due to vascular involvement of the tumor (vascular R1), and 38 (71.7%) were unintentional (parenchymal) R1 resections. The ICG fluorescence was observed in the transection plane during 59 CRLM resections. After correlation with histopathologic results, the ICG-fluorescence imaging for intraoperative tumor margin assessment led to a sensitivity and specificity of ICG-fluorescence imaging for intraoperative tumor margin assessment of 60% and 90%, respectively (Table 2). The PPV (presence of ICG-fluorescence signal for an R1 resection) was 54%, while the NPV (absence of ICG fluorescence for an R0 resection) was 92%. The false-positive rate of fluorescence was 41% (11 of 27). The area under the receiver operating characteristic curve was 0.751 (95% CI, 0.668-0.833) (Figure 3). After excluding patients who received neoadjuvant therapy, sensitivity was 62% and specificity was 91%, whereas the PPV was 61%, and the NPV was 91%. eFigure 1 in Supplement 1 provides a graphical representation of the anatomical distribution of the resected CRLM.

**Table 2.** Imaging Accuracy Contingency for Number of Colorectal Liver Metastases

Imaging	Resections, No. (%)			
	Vascular R1	Parenchymal R1	R0	Total
NIR+^a^	14 (23.7)	18 (30.5)	27 (45.8)	59
NIR−^b^	1 (0.4)	20 (7.8)	236 (91.8)	257
Total	15 (4.7)	38 (12.0)	263 (83.2)	316

Abbreviations: NIR, near-infrared; R0, negative tumor margin; R1, positive tumor margin.

^a^ Positive NIR fluorescence signal.

^b^ Negative NIR fluorescence signal.

### Changes in Surgical Management

Overall, the use of ICG fluorescence was associated with a change in surgical management in 56 (27.9%) of 201 patients. During 56 (17.7%) of 316 resections, the surgeon adapted the transection plane based on ICG fluorescence to increase the resection margin. In 27 resections (8.5%), additional tissue was resected as mentioned above.

A total of 38 newly diagnosed ICG-fluorescence–positive additional lesions suspected of being occult CRLM were resected in 29 patients; 26 (68.4%) were confirmed CRLM after histopathologic assessment (eTable 3 in Supplement 1). On a patient level, this led to the identification of additional CRLM in 19 (9.5%) of 201 patients. The sensitivity and specificity of ICG-fluorescence for the detection of additional CRLM were 90% and 27%, respectively, with a PPV of 70% and an NPV of 57% (area under the receiver operating characteristic curve, 0.583; 95% CI, 0.399-0.769) (eFigure 2 in Supplement 1).

### Learning Curve

All surgeons participating in the study returned the questionnaire (eAppendices 1 and 2 in Supplement 1). Two of the 8 participating centers (4 surgeons) had previous experience with ICG fluorescence to identify liver lesions and to assess resection margins and were considered experienced surgeons. The remainder of the surgeons were trained prior to study initiation as detailed above (eAppendix 3 in Supplement 1). All inexperienced and experienced surgeons perceived that they went through a learning curve. The most important items in the learning curve were to be able to differentiate malignant lesions from benign ICG-fluorescence–positive lesions and to predict R1 resections based on ICG fluorescence. Experienced centers (>10 procedures) scored similarly compared with inexperienced centers (≤10 procedures) in interpreting residual ICG fluorescence to predict an R1 resection (odds ratio, 1.36; 95% CI, 0.44-4.24). Thereafter, the resection margin status of experienced centers was compared with inexperienced centers. An R1 rate of 21.7% was found for inexperienced centers and 14.7% for experienced centers (*P* = .13) (eTable 3 in Supplement 1). The odds of differentiating true-positive additional lesions from false-positive additional lesions were higher in experienced vs nonexperienced centers, but this result was not significant (odds ratio, 1.75; 95% CI, 0.41-7.45). Importantly, 89% of participating surgeons continued using ICG fluorescence for minimally invasive CRLM surgery as the standard of care after study completion.

### Perioperative Complications

No adverse events regarding the injection of ICG were documented. The median operative blood loss was 150 mL (IQR, 50-350 mL). Severe (Clavien-Dindo grades 3 to 5; range 0-5) complications occurred in 11 patients and were not related to ICG injection, intraoperative imaging, or additional resection. No patients died in the first 30 days after surgery. The median length of stay was 3 days (IQR, 2-4 days) (Table 1).

## Discussion

In the single-arm prospective multicenter cohort MIMIC trial, we found that ICG fluorescence was associated with an increase in the R0 resection rate to an overall 92.4%. Negative ICG fluorescence in the parenchymal transection plane was highly accurate (92%) in predicting a margin-negative resection. Moreover, real-time fluorescence assessment of the resection cavity provided the opportunity to perform a direct re-resection in patients with an ICG-fluorescence–positive resection specimen, ultimately increasing radical resection rates. Overall, we found that in more than one-quarter (27.9%) of the procedures, ICG fluorescence led to an alteration of the surgical plan.

We also found that the use of ICG fluorescence resulted in the resection of additional tissue in a substantial number (8.5%) of patients, leading to an increase in radical resection rates. Yet, the long-term oncologic benefit for the patients in whom additional tissue was resected remains uncertain and can only be concluded after long-term follow-up of this cohort. Although the 1-mm resection margin is currently considered the gold standard for an R0 resection, this is not without debate.^25,26,27,28,29,30^ Some retrospective studies have suggested that tumor debulking (0 mm margin) might suffice, whereas in some cases, more margin is required to avoid local tumor recurrence,^26,31^ especially for *KRAS*-mutated primary tumors. Specifically for CRLM, consensus molecular subtyping was studied in 2018 and suggested that different risk patterns exist which define a biologic basis for curing patients with CRLM.^32^ Empirically, if more than only tumor cells dictate the behavior of a CRLM and vary among patients, the importance of a resection margin is dictated by the biologic basis (eg, mutation status, histopathologic growth pattern) of the tumor irrespective of the anatomical location.^33^ In this perspective, tumor-specific fluorescent contrast agents designed to interact with overexpressed tumor receptors or immune cells in the stroma have already been shown to be feasible in primary colorectal cancer.^34,35,36^

Implementing ICG-fluorescence imaging outside centers of expertise is thought to be the major challenge for the adoption of ICG-fluorescence as a standard-of-care imaging procedure. Our data suggest that implementation is possible by training surgical personnel and by providing preset imaging protocols. Interestingly, for inexperienced centers, the percentage of false-positive additional lesions in the first 10 procedures was higher compared with later procedures. Also, the results of the questionnaire provided after study completion suggest that ICG fluorescence for minimally invasive CRLM surgery was not plug and play. Both data points show that surgeons experienced a learning curve of some extent. Hence, careful introduction by means of providing course materials and proctoring (or teleproctoring) of surgeons willing to get acquainted with the technique appears to be a feasible approach to expand the use of ICG-fluorescence–guided surgery. This was also supported by our experience that all participating surgeons are willing to participate in future studies, and 89% of respondents still use ICG for fluorescence-guided liver surgery after study completion.

The use of neoadjuvant treatment for patients with CRLM is relatively limited in the Netherlands and only indicated for patients who are initially deemed not resectable. In our cohort, 28.9% of all patients included in the study received neoadjuvant chemotherapy, which is in line with most recent American Society of Clinical Oncology recommendations.^37^ Some of the surgeons reported in the eCRF that interpretation of ICG-fluorescence imaging was more challenging in patients pretreated with chemotherapy; however, this was not confirmed by the objective data in our study population that found similar sensitivities and specificities in both groups. Accordingly, current Asia-Pacific consensus guidelines also suggest that signal interpretation can be more difficult, and ICG administration should be altered in this patient group to prevent high false-positive rates.^38^ However, in line with current literature,^39,40^ the initial radical resection rates per lesion were comparable for patients treated with neoadjuvant chemotherapy (86%) vs those who were not pretreated with chemotherapy (81%) (*P* = .35).

### Limitations

A limitation of this study is its single-arm design, which prevents further comparison between cohorts. However, we feel that such an implementation study should be initially performed single arm for proper exposure of the technique to the participating clinicians. Further research can now be done in a randomized clinical trial, looking into cost-benefit and patient variables rather than lesion variables. Second, no real-time quantification method for fluorescence for use in the clinic was available, and it may be that quantitative results would be superior to the qualitative method that we used. A subanalysis comparing the imaging systems did not provide enough power for a proper conclusion. Yet, with the available data, there was no difference in imaging accuracy. From a surgeon’s perspective, this real-time surgical-margin feedback during parenchymal transection may help in achieving tumor-negative margins; however, alterations of the transection path during resection were difficult to correlate to margin negativity since these alterations were not considered during pathological evaluation.

We found no difference in the accuracy of residual ICG fluorescence for the detection of nonradical resections between the camera systems. However, it may be that the use of several clinically available laparoscopic systems with distinctive NIR fluorescent lasers and settings is not ideal. On the other hand, the absence of large differences could also be indicative of a minor contribution of this issue. This would imply that minimally invasive ICG-fluorescence–guided liver surgery could be implemented broadly without requiring the use of one specific laparoscope. Finally, no control group was used in this study with which the primary outcome could be compared.

## Conclusions

The findings of this prospective multicenter cohort study suggest that ICG-fluorescence imaging may increase radical resection rates in minimally invasive surgery for CRLM. More specifically, the absence of an ICG-fluorescence signal predicted a radical resection with 92% certainty. In addition, the data showed that ICG-fluorescence imaging led to an alteration of a treatment plan in more than one-quarter of patients, suggesting that this procedure may provide surgeons with real-time feedback of tumor margins.

## Notes

Supplement 1. **eTable 1.** Definitions Used for the Contingency Table for the Accuracy Analysis **eTable 2.** Baseline Table Displaying Similar Preoperative, Intraoperative, and Postoperative Characteristics of Patients and Lesion Data in Both the Laparoscopy Group and the Robot-Assisted Surgery Group **eTable 3.** Learning Curve-Related Outcome in Which a Center Was Deemed Experienced After 10 Procedures **eFigure 1.** Graphical Representation of the Anatomical Distribution, According to the Couinaud Classification of the 316 Histopathologically Confirmed Colorectal Liver Metastases (CRLM) **eFigure 2.** ROC Curve of the Accuracy of ICG-Fluorescence Imaging for the Intraoperative Detection of Additional Lesions **eAppendix 1.** Questionnaire (Original) **eAppendix 2.** Questionnaire (English Translation) **eAppendix 3.** Link to the Editorial Video Used for Training
eReference

Supplement 2.
Data Sharing Statement
