# Supplementary material for: ICG-Fluorescence Imaging for Margin Assessment During Minimally Invasive Colorectal Liver Metastasis Resection
Source: JAMA Netw Open. 2024 Apr 19;7(4):e246548. doi: 10.1001/jamanetworkopen.2024.6548 (PMC11031680; doi:10.1001/jamanetworkopen.2024.6548)
Supplement: Supplement 1. — eTable 1. Definitions Used for the Contingency Table for the Accuracy Analysis eTable 2. Baseline Table Displaying Similar Preoperative, Intraoperative, and Postoperative Characteristics of Patients and Lesion Data in Both the Laparoscopy Group and the Robot-Assisted Surgery Group eTable 3. Learning Curve-Related Outcome in Which a Center Was Deemed Experienced After 10 Procedures eFigure 1. Graphical Representation of the Anatomical Distribution, According to the Couinaud Classification of the 316 Histopathologically Confirmed Colorectal Liver Metastases (CRLM) eFigure 2. ROC Curve of the Accuracy of ICG-Fluorescence Imaging for the Intraoperative Detection of Additional Lesions eAppendix 1. Questionnaire (Original) eAppendix 2. Questionnaire (English Translation) eAppendix 3. Link to the Editorial Video Used for Training eReference [file jamanetwopen-e246548-s001.pdf]

Supplemental Online Content

Achterberg FB, Bijlstra OD, Slooter MD, et al. ICG-fluorescence imaging for margin assessment during minimally invasive colorectal liver metastasis resection. *JAMA Netw Open*. 2024;7(4):e246548. doi:10.1001/jamanetworkopen.2024.6548

eTable 1. Definitions Used for the Contingency Table for the Accuracy Analysis
eTable 2. Baseline Table Displaying Similar Preoperative, Intraoperative, and Postoperative Characteristics of Patients and Lesion Data in Both the Laparoscopy Group and the Robot-Assisted Surgery Group
eTable 3. Learning Curve-Related Outcome in Which a Center Was Deemed Experienced After 10 Procedures
eFigure 1. Graphical Representation of the Anatomical Distribution, According to the Couinaud Classification of the 316 Histopathologically Confirmed Colorectal Liver Metastases (CRLM)
eFigure 2. ROC Curve of the Accuracy of ICG-Fluorescence Imaging for the Intraoperative Detection of Additional Lesions
eAppendix 1. Questionnaire (Original)
eAppendix 2. Questionnaire (English Translation)
eAppendix 3. Link to the Editorial Video Used for Training
eReference

This supplemental material has been provided by the authors to give readers additional information about their work.

© 2024 Achterberg FB et al. *JAMA Network Open*.

eTable 1. Definitions Used for the Contingency Table for the Accuracy Analysis

	**Histologically proven R1**	**Histologically proven R0**
**ICG-fluorescence +**	True positive	False positive
**ICG-fluorescence -**	False negative	True negative

© 2024 Achterberg FB et al. *JAMA Network Open*.

eTable 2. Baseline Table Displaying Similar Preoperative, Intraoperative, and Postoperative Characteristics of Patients and Lesion Data in Both the Laparoscopy Group and the Robot-Assisted Surgery Group

**Patient characteristics**	**Cohort** (n=201)	**Laparoscopy** (n= 71)	**Robot-assisted** (n= 130)	**P-value**
Age, years (median, IQR)	65 (57 – 73)	64	64	0.681
Sex (male, %)	112 (57)	36	76	0.231
CEA (ng/mL) (median, IQR)	5.7 (2.55-15)	23.0	19.7	0.787
Number of suspected CRLM (mean, sd)	2.2 (2.3)	2.3	2.1	0.973
Neoadjuvant chemotherapy (%)	58 (29)	26	32	0.076
**Surgery characteristics**	**Cohort**	**Laparoscopy**	**Robot-assisted**	
Operation time, mins (mean, sd)	175 (80)	185	170	0.643
Estimated blood loss, mL (mean, sd)	315 (589)	358	293	0.338
Hospital stay, days (median, IQR)	3 (2 - 4)	3.7	3.4	0.831
Clavien-Dindo classification				0.398
None (0)	169	61	108	
Low (1-2)	15	3	12	
High (3-5)	11	3	8	
Initial resection margin				0.443
R0 (%)	263 (83)	99 (85)	164 (82)	
R1 (%)	53 (17)	17 (15)	36 (18)	
Final resection margin				0.937
R0 (%)	280 (89)	103 (89)	177 (89)	
R1 (%)	36 (11)	13 (11)	23 (11)	

© 2024 Achterberg FB et al. *JAMA Network Open*.

eTable 3. Learning Curve-Related Outcome in Which a Center Was Deemed Experienced After 10 Procedures

**Outcome**	**Experienced center** (number of resections, (%))	**Inexperienced center** (number of resections, (%))	**P-value**
Initial resection margin status			0.130
R0	191 (85.3)	72 (78.3)	
R1	33 (14.7)	20 (21.7)	
Final resection margin status			0.268
R0	202 (90.2)	79 (85.9)	
R1	22 (11.4)	13 (14.1)	
Additional NIR + lesions resected	17	20	0.447
True positive	13 (76.5)	13 (65.0)	
False positive	4 (24.5)	7 (35.0)	

© 2024 Achterberg FB et al. *JAMA Network Open*.

eFigure 1. Graphical Representation of the Anatomical Distribution, According to the Couinaud Classification of the 316 Histopathologically Confirmed Colorectal Liver Metastases (CRLM)

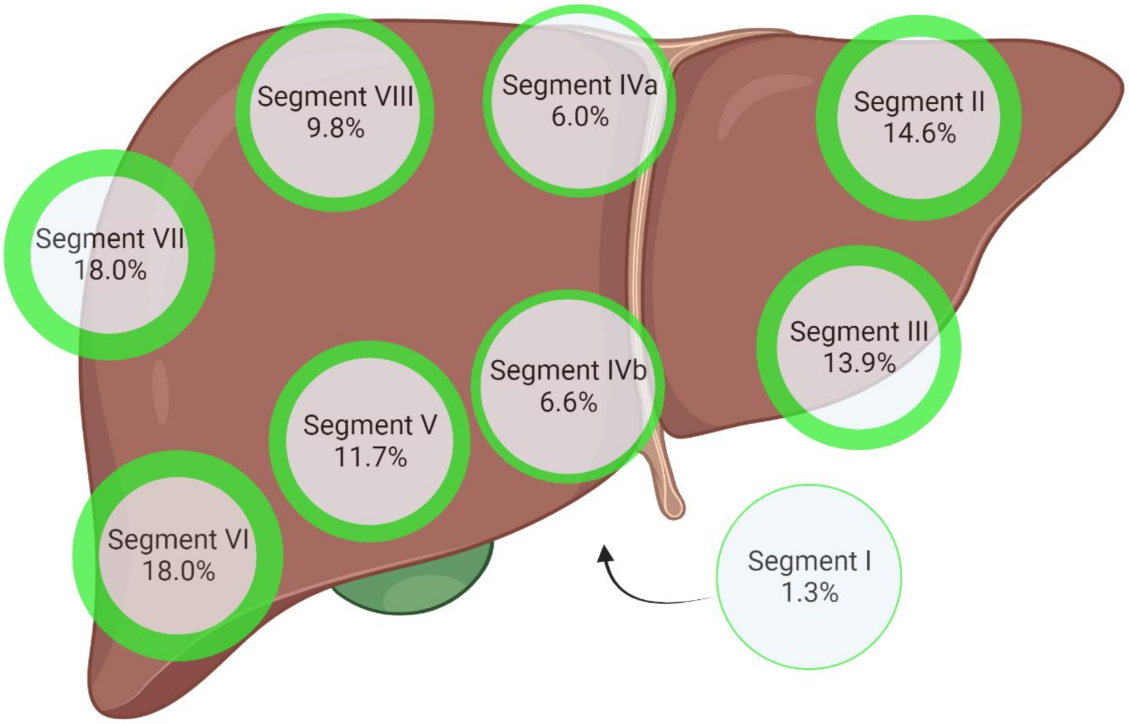

The rim width is indicative of the number of CRLM resected from a liver segment.

© 2024 Achterberg FB et al. *JAMA Network Open*.

eFigure 2. ROC Curve of the Accuracy of ICG-Fluorescence Imaging for the Intraoperative Detection of Additional Lesions

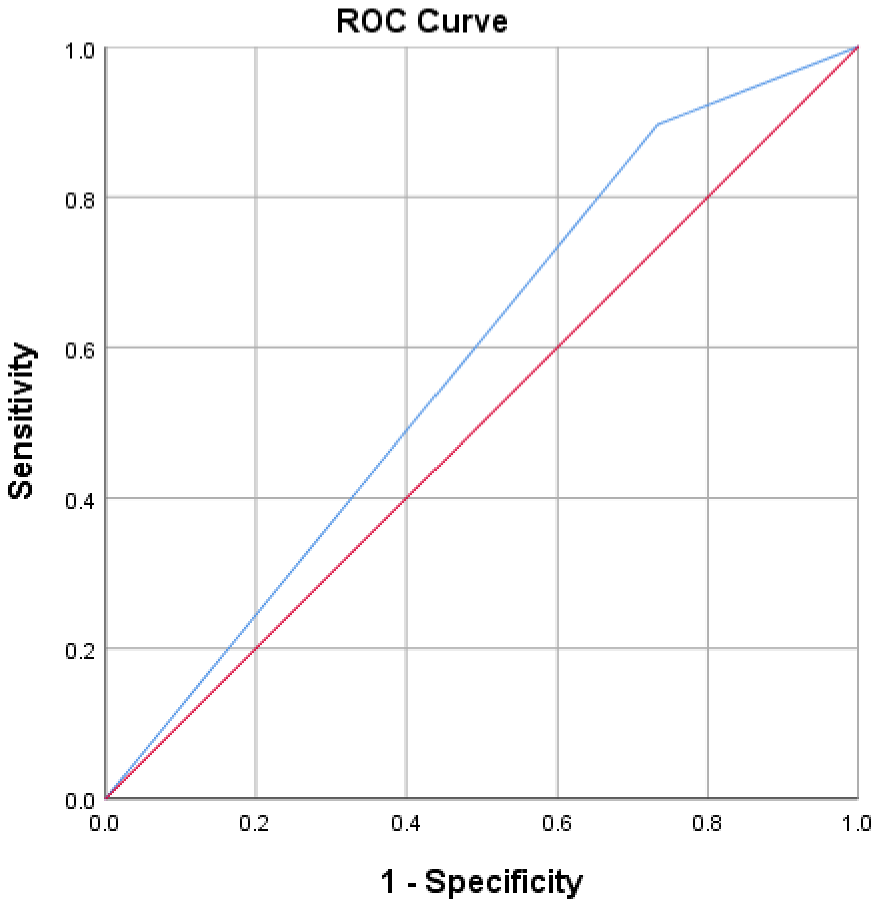

© 2024 Achterberg FB et al. *JAMA Network Open*.

eAppendix 1. Questionnaire (Original)

1. Hoeveel laparoscopische- en robot-geassisteerde leverresecties worden er jaarlijks verricht in uw centrum volgens de DHBA database?

- Laparoscopisch:

- Robot-geassisteerd:

1a. Wat is daarvan het percentage voor CRLM?

2. Bij hoeveel van de mininmaal-invasieve leverresecties in uw centrum maakt u gebruikt van indocyanine groen voor het opsporen van tumoren en/of het bepalen van uw resectiemarge?

a) alles

b) alleen in geselecteerde gevallen, namelijk: …………..

3. Gebruikte u al indocyanine groen vóór uw deelname aan de MIMIC-studie? Zo ja, hoelang al?

3a. Heeft u een leercurve moeten doormaken voordat u zich comfortabel en vertrouwd voelde met het gebruik van fluorescentie-geleide leverchirurgie met ICG?

4. Waar zat de leercurve met name in?
a. Herkennen target laesie / fluorescentiepatroon

b. Identificeren additionele laesie(s)

c. Beoordeling resectiemarges

d. subjectieve kwantificatie van fluorescentiesignaal, tov het omliggende weefsel

e. alle bovenstaande antwoorden

f. anders, nl…

5. Neemt u extra leverweefsel weg, indien de er fluorescentie te zien is in het resectievlak of de resectieholte?

© 2024 Achterberg FB et al. *JAMA Network Open*.

6. Voor een additionele laesie met een “ring fluorescentie patroon” doe ik:

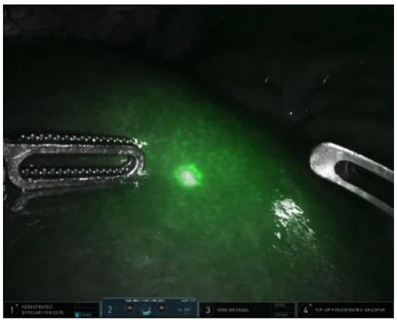

- *a) een vriescoupe*
- *b) oncologische (wig)resectie*
- *c) geen resectie*
- *d) anders, nl…*

7. Voor een additionele laesie die “geheel fluorescent is” doe ik:

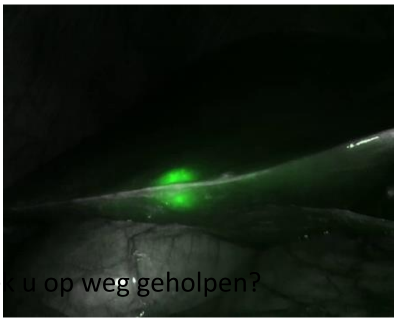

- *a) een vriescoupe*
- *b) oncologische (wig)resectie*
- *c) geen resectie*
- *d) anders, nl…*

8. Heeft de uitleg/de training die u kreeg voorafgaand aan het onderzoek u op weg geholpen?

9. Gebruikt u nu (na sluiting van de MIMIC) nog steeds indocyanine groen tijdens leverchirurgie?

10. Waarin zit voor u de meeste meerwaarde in het gebruik van fluorescentie-geleide chirurgie met indocyanine groen?

11. Wat vindt u de grootste tekortkoming(en) aan deze techniek?

12. Hoe is de logistiek bij u geregeld rondom fluorescentie-geleide chirurgie met betrekking tot de injectie van indocyanine groen en de camerasystemen?

13. Heeft u interesse in deelname aan een eventuele vervolgstudie van de MIMIC?

© 2024 Achterberg FB et al. *JAMA Network Open*.

eAppendix 2. Questionnaire (English Translation)

How many laparoscopic and robot assisted liver resections are being performed annually in your hospital according to the DHBA (Dutch hepatobiliary audit)?

- Laparoscopic:
- Robot assisted:

1a. In what percentage does it apply surgery for CRLM?

1. In what number of cases do you already use indocyanine green (ICG) for the detection of occult liver metastases and/or determining the resection margin?
   a) In all cases
   b) Only in selected cases, namely:…..
   c)

2. Did you have any experience with using indocyanine green prior to participation of the MIMIC Trial? If so, for how long?

   3a. Did you experience a learning-curve before confidently using fluorescence-guided for liver surgery?

4. In what area did you experience the learning-curve?
a. Recognizing the fluorescence pattern/lesion

b. Identifying occult liver metastases

c. Assessing the resection margin

d. subjective quantification of the fluorescence signal in relation to the surrounding tissue

e. all of the above

f. Different, namely:…

5. Do you perform an additional resection of the surgical cavity if fluorescence is present in the cavity?

6. In case you identify a fluorescent lesion with a 'rim' pattern, what would you do?

a) Fresh frozen section
b) Oncological wedge resection

c) No resection

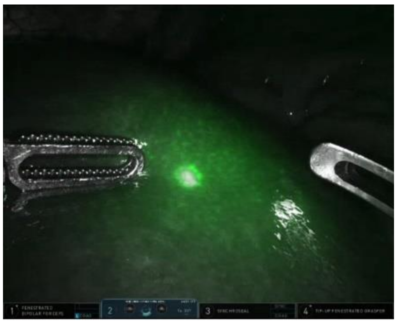

© 2024 Achterberg FB et al. *JAMA Network Open*.

d) *Something different, namely:…*

7. In case you identify a complete fluorescent lesion, what would you do?

a) *Fresh frozen section*
b) *Oncological wedge resection*
c) *No resection*
d) *Something different, namely:…*

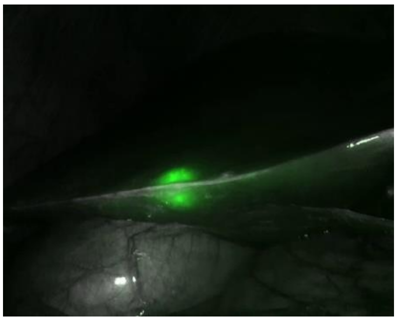

8. Did the provided training material/instructional movie help with your learning-curve?

9. Do you (after completing the MIMIC Trial) still use ICG for liver resections?

10. Where does, according to your opinion, fluorescence-guided surgery for patients with CRLM aid the most?

11. If so, what would be a downside of using ICG?

12. How does you department organize the intravenous injection of ICG 24 hours prior to surgery?

13. Would your department be interested in participating in any follow-up/future studies?

© 2024 Achterberg FB et al. *JAMA Network Open*.

eAppendix 3. Link to the Editorial Video Used for Training

Link to the video:

Real-time surgical margin assessment using ICG-fluorescence during laparoscopic and robot-assisted resections of colorectal liver metastases - Achterberg - Annals of Translational Medicine (amegroups.org)

© 2024 Achterberg FB et al. *JAMA Network Open*.

eReference

Achterberg FB, Sibinga Mulder BG, Meijer RPJ, Bonsing BA, Hartgrink HH, Mieog JSD, Zlitni A, Park Sm, Farina Sarasqueta A, Vahrmeijer AL, Swijnenburg RJ. Real-time surgical margin assessment using ICG-fluorescence during laparoscopic and robot-assisted resections of colorectal liver metastases. Ann Transl Med 2020;8(21):1448. doi: 10.21037/atm-20-1999.

© 2024 Achterberg FB et al. *JAMA Network Open*.
